# Supplementary material for: Enhanced multiscale human brain imaging by semi-supervised digital staining and serial sectioning optical coherence tomography
Source: Light Sci Appl. 2025 Jan 20;14:57. doi: 10.1038/s41377-024-01658-0 (PMC11746934; doi:10.1038/s41377-024-01658-0)
Supplement: Supplementary file 1 — Supplementary materials [file 41377_2024_1658_MOESM1_ESM.pdf]

Supplementary Information for

Enhanced Multiscale Human Brain Imaging by Semi-supervised Digital Staining and Serial Sectioning Optical Coherence Tomography

Authors

Shiyi Cheng[1†], Shuaibin Chang[1†], Yunzhe Li[2†], Anna Novoseltseva[3], Sunni Lin[1,3], Yicun Wu[4], Jiahui Zhu[1], Ann C. McKee[5,6,7,8], Douglas L. Rosene[9], Hui Wang[10], Irving J. Bigio[1,3,11], David A. Boas[1,3,11], Lei Tian[1,3,11,*]

Affiliations

[1] Department of Electrical and Computer Engineering, Boston University, Boston, MA, 02215, USA.

[2] Department of Electrical Engineering and Computer Sciences, University of California, Berkeley, CA, 94720, USA.

[3] Department of Biomedical Engineering, Boston University, Boston MA, 02215, USA.

[4] Department of Computer Science, Boston University, Boston, MA, 02215, USA.

[5] Boston University Alzheimer's Disease Research Center and CTE Center, Boston University School of Medicine, Boston, MA, 02118, USA.

[6] Department of Neurology, Boston University School of Medicine, Boston, MA, 02118, USA.

[7] VA Boston Healthcare System, U.S. Department of Veteran Affairs, Boston, MA, 02130, USA.

[8] Department of Pathology and Laboratory Medicine, Boston University School of Medicine, Boston, MA, 02118, USA.

[9] Department of Anatomy & Neurobiology, Boston University School of Medicine, Boston, MA, USA.

[10] Department of Radiology, Athinoula A. Martinos Center for Biomedical Imaging, Massachusetts General Hospital, Boston, MA, 02129, USA

[11] Neurophotonics Center, Boston University, Boston, MA, 02215, USA.

[†]These authors contributed equally to this work.

[*]**Email:** leitian@bu.edu [*]**Telephone**: (617)353-1334

1. Overall qualitative comparisons between traditional physical staining, S-OCT and our DS-OCT techniques

Generally, our proposed DS-OCT method significantly enhances the interpretability of S-OCT while preserving the benefits of high volumetric imaging throughput and uniform staining, compared to traditional physical staining methods, as qualitatively illustrated in Figure S1. It is important to note that various metrics can be used to quantify each imaging attribute. We have attempted to quantify them using multiple metrics, detailed in the main text and supplementary materials in Sections 5, 7, 13, 14, 15, and 16.

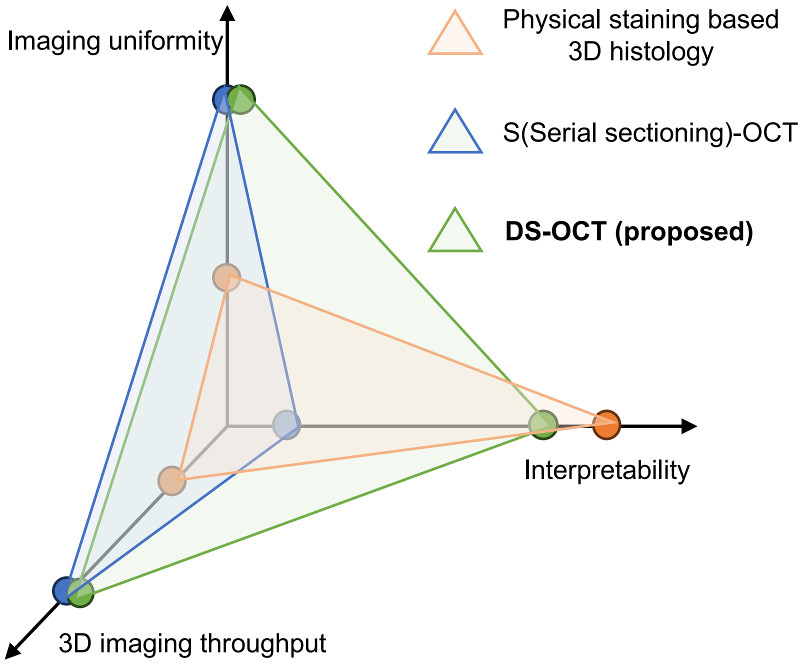

Figure S1. Overview of comparisons between traditional physical staining, S-OCT and our DS-OCT techniques. Our proposed DS-OCT method largely enhances the interpretability of S-OCT while maintaining the advantages of high volumetric imaging throughput and staining uniformity over traditional physical staining methods.

2. Qualitative comparisons against previous baseline models

Compared to previous fully supervised or fully unsupervised methods, our semi-supervised approach exploits the side information from bio-physical model to provide pseudo-supervised learning module, which has the advantage of minimizing hallucination (de-hallucination). It additionally exploits information from adjacent slices to learn a co-registration learning module, which facilitates pair-wise image evaluations, as summarized in Table S1. Note that we mark CUT as successful at de-hallucination (using self-supervision) as well since the analysis in Figure S5 shows slight differences and is pending future validation.

Advantages	De-hallucination	Co-registration	No need for paired data	Pair-wise evaluation
Supervised	✓	N.A.	✗	✓
CycleGAN	✗	✗	✓	✗
CUT/FastCut	✓	✗	✓	✗
**Ours**	**✓**	**✓**	**✓**	**✓**

Table S1. Advantages comparison of our proposed semi-supervised methods to previous methods for digital staining.

We evaluate our semi-supervised approach against several baseline methods. Figure S2 shows whole slide images (WSIs) of OCT-SC, DS with four training methods and PS images from adjacent cortex sections. The baseline methods are Supervised[1], CycleGAN[2], CUT and FastCUT models[3]. Quantitative evaluations are provided in Section 3. In addition, Section 4 explains their training details. Our model outperforms them in resolution and color contrast similarity to PS images, as seen in the whole field-of-view (FOV) and zoom-in regions of interest (ROIs).

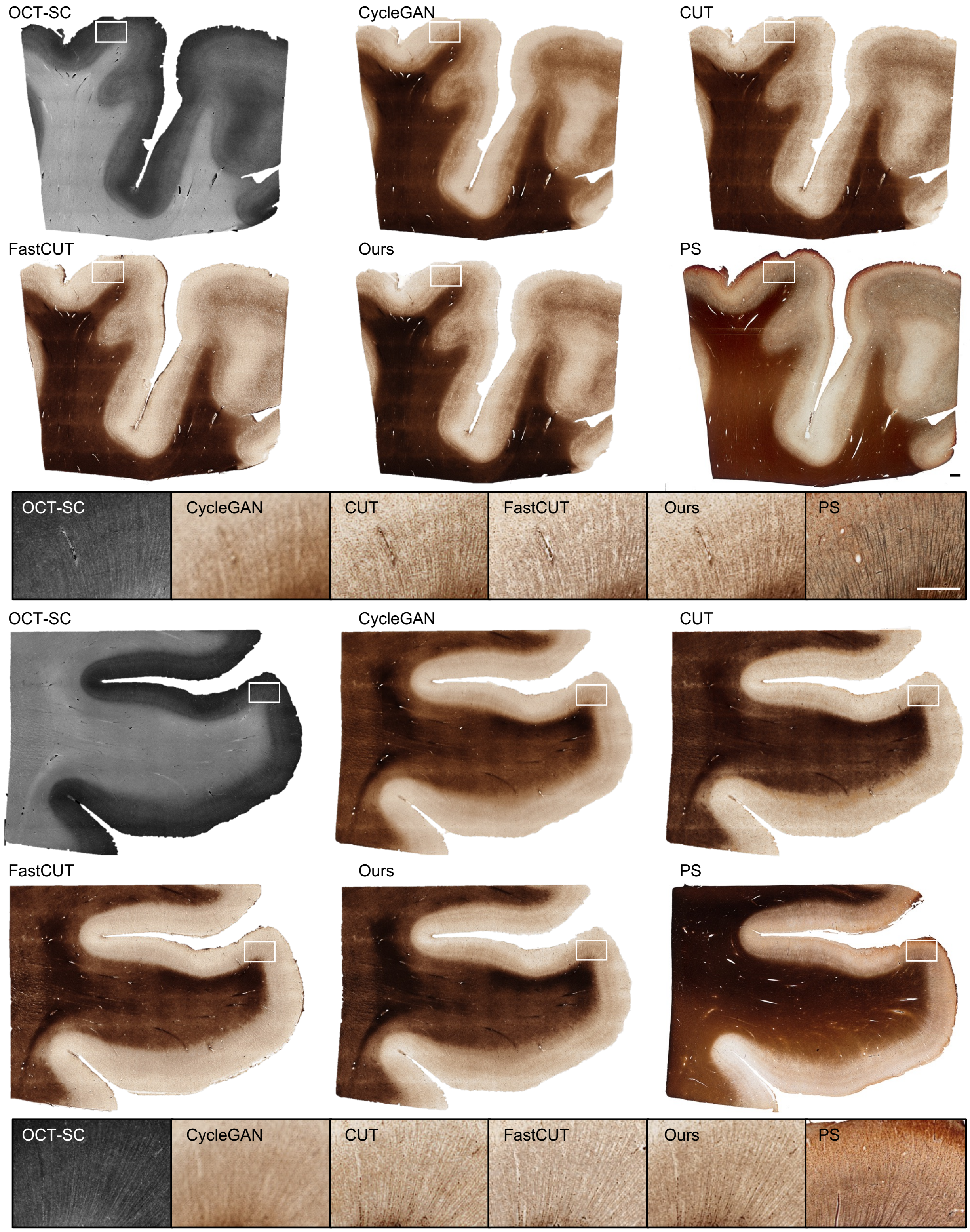

Figure S2. Qualitative comparisons against previous baseline models. Results from four methods are visualized: CycleGAN, CUT, FastCUT, and Ours. PS images from adjacent cortex tissue sections are shown as references. Zoom-in regions are marked by the white rectangular boxes in WSIs. Scale bars are 1 mm.

3. Quantitative comparisons to baseline models

We present the quantitative comparison results between our approach and other baseline methods in Figure S4. To address the challenges posed by the weakly paired dataset, we have devised custom quantitative metrics for both pairwise (by leveraging our trained registration network module, see Figure S3) and unpaired evaluations. Detailed descriptions of the training parameter settings of deep learning models and quantitative metrics we employ can be found in Section 4 and Section 5, respectively. These metrics are visualized using box plots, which indicate the median and quantile values, employing five distinct colors to represent four different models and the PS images.

Figure S3 illustrates the generation of registered pairs using our cross-modality registration model R. To quantitatively evaluate the DS image metrics compared to PS images, in Figure S3A, we use the trained registration network to generate coarsely registered pairs of DS and PS. In Figure S3B, we can see the image correlation score (Pearson correlation coefficient) rises from around 0.6 to around 0.9 after image registration.

In Figure S4A, we focus on pairwise metrics comparing registered PS images and DS images within the well-stained sample group to ensure fair comparisons. Lower values are desired for metrics such as mean squared error (MSE) and color difference (CD), while higher values are preferred for metrics such as Pearson correlation coefficient (PCC) and intersection over union of layers (IOU-layers). Among the four pairwise metrics, our method consistently outperforms three baseline models, exhibiting better scores and smaller variations. Note by the standard metrics, CUT and our method are almost comparable in terms of average score, indicating that customized feature-based metrics are needed to enable finer analysis.

Moving on to Figure S4B, we design unpaired metrics that evaluate either the per-image distribution or the distribution of the entire dataset. These metrics are applied to samples across the entire sample group, with PS images remaining unregistered (to avoid image feature distortion). We extract myelin fiber (MF) segments from the DS and (adjacent) PS images, computing histograms of different properties for comparison, such as fiber segment lengths (MF length) and fiber segment diameter (MF diameter). The divergence between the histograms of these properties is quantified using Jensen-Shannon divergence (JS). "JS-MF length" corresponds to Jensen-Shannon divergence (JS) of myelin fiber segment (MF) lengths, while "JS-MF diameter" refers to JS of myelin fiber segment diameters. Lower values are desired for both JS-MF length and JS-MF diameter. Our proposed model outperforms CycleGAN in both metrics, performs comparably to CUT and FastCUT in terms of JS-MF length, but excels in terms of JS-MF diameter.

Additionally, we compute the total number of myelin fiber segments within each sample image (MF-segments number) and the percentage of area occupied by the fiber segments (MF-area percentage) based on the extracted myelin fiber segments. These two metrics are used to analyze the distributions across the entire sample group, regardless of staining quality, to capture the variability of each method. Our method exhibits metric distributions similar to FastCUT, showcasing comparable performance to PS images. Notably, our method demonstrates the closest median value to that of the PS images while reducing variations. Overall, both the paired and unpaired quantitative metrics presented in this section demonstrate the superior performance of our method compared to other baseline methods.

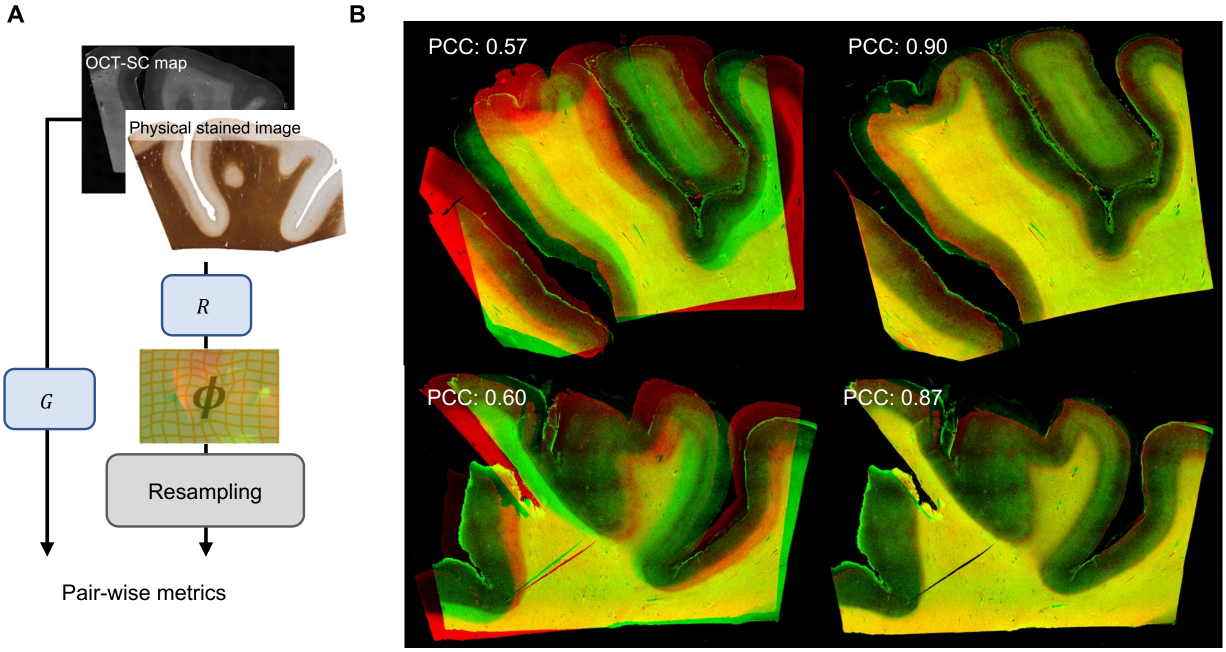

Figure S3. Pairwise metrics computation on registered image pairs. (A) The DS images are generated by feeding the SC images to the DS model. The original PS images are resampled by the deformation field output from the registration network through image warping. Those registered pairs are later used for pairwise metric evaluation. (B) The left column shows unregistered grayscale images in green and red channels, which are converted and normalized from SC and PS images, respectively. The right column shows the same image pairs after PS images are registered. Pearson correlation coefficients (PCC) are shown to indicate image alignment score.

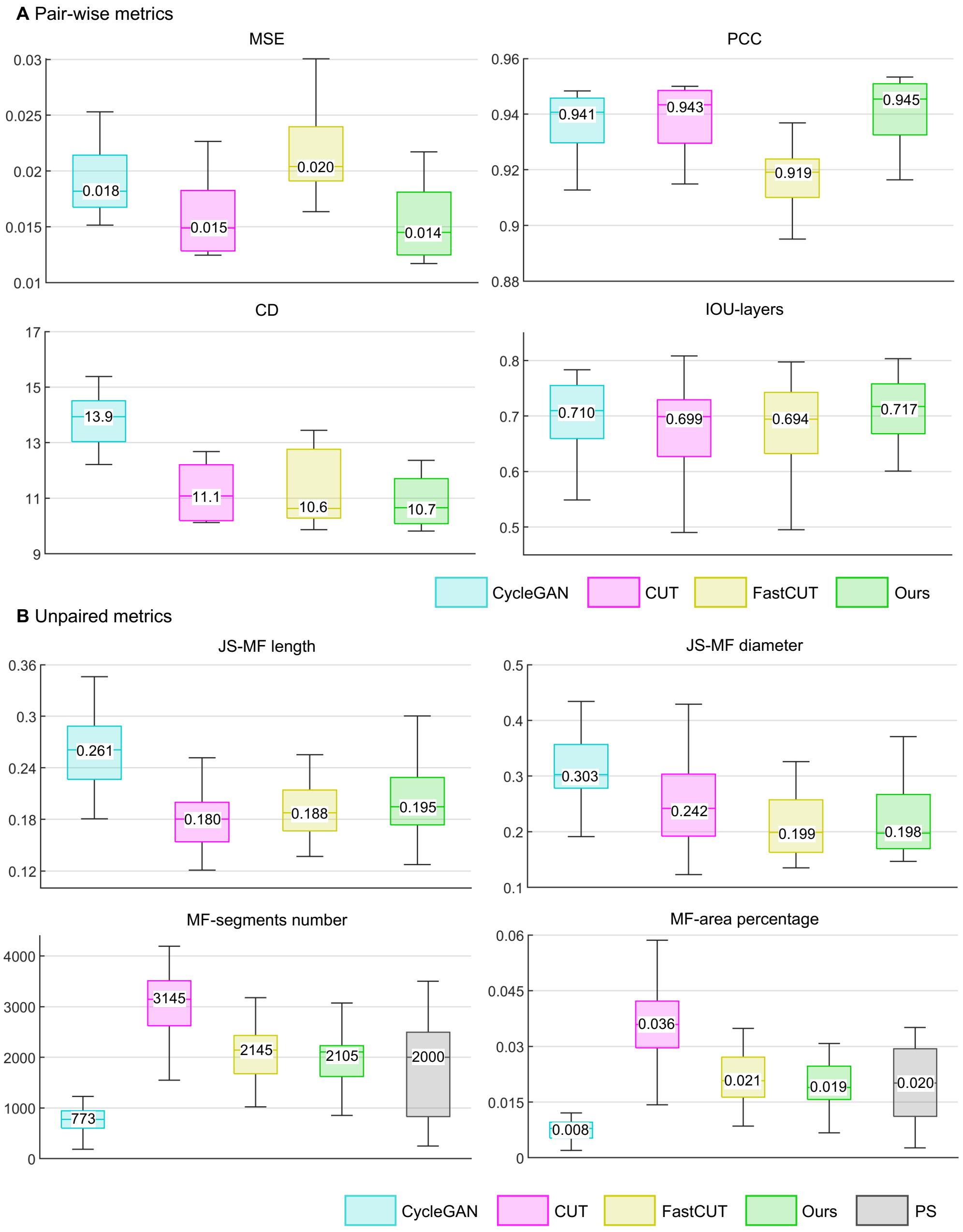

Figure S4. Quantitative comparisons with baseline methods. (A) Box plots illustrate four pairwise metrics, each represented by a unique color denoting the compared methods. The metrics include mean squared error (MSE) and color difference (CD), with lower values indicating better performance. Additionally, Pearson correlation coefficient (PCC) and intersection over union score of extracted layer IV/V/VI (IOU-layers) are included, where higher values are desired for improved results. The embedded numbers within the plots represent the median values for each metric. (B) Box plots present four unpaired metrics in five colors, with an additional gray color representing the metrics derived from PS images. Lower JS scores indicate superior performance for both metrics. "MF-segments number" and "MF-area percentage" display the distribution of reference-less metrics across the entire dataset. Closer alignment to the metrics obtained from PS images is indicative of better results.

To further provide a quantitative metric of model hallucination, we propose an application specific evaluation pipeline and compute a fidelity score, as illustrated in Figure S5. The main idea is based on our biophysical model that a linear correlation between the OCT scattering coefficients (SC) and the optical density (OD) computed from the Gallyas silver stained image [4]. We argue that a high-fidelity digitally stained Gallyas silver image should remain the same linear correlation. By applying this argument, we quantify the fidelity by evaluating the correlation between the OD derived from the DS-stained image and the *physical* OCT-SC map. In Figure S5, we computed the fidelity scores of four DS methods: CycleGAN, CUT, our semi-supervised framework, and pseudo-supervised method. With the strength of supervision ranging from low to high, the DL model is expected to generate less hallucinations, which our evaluation results agreed well with.

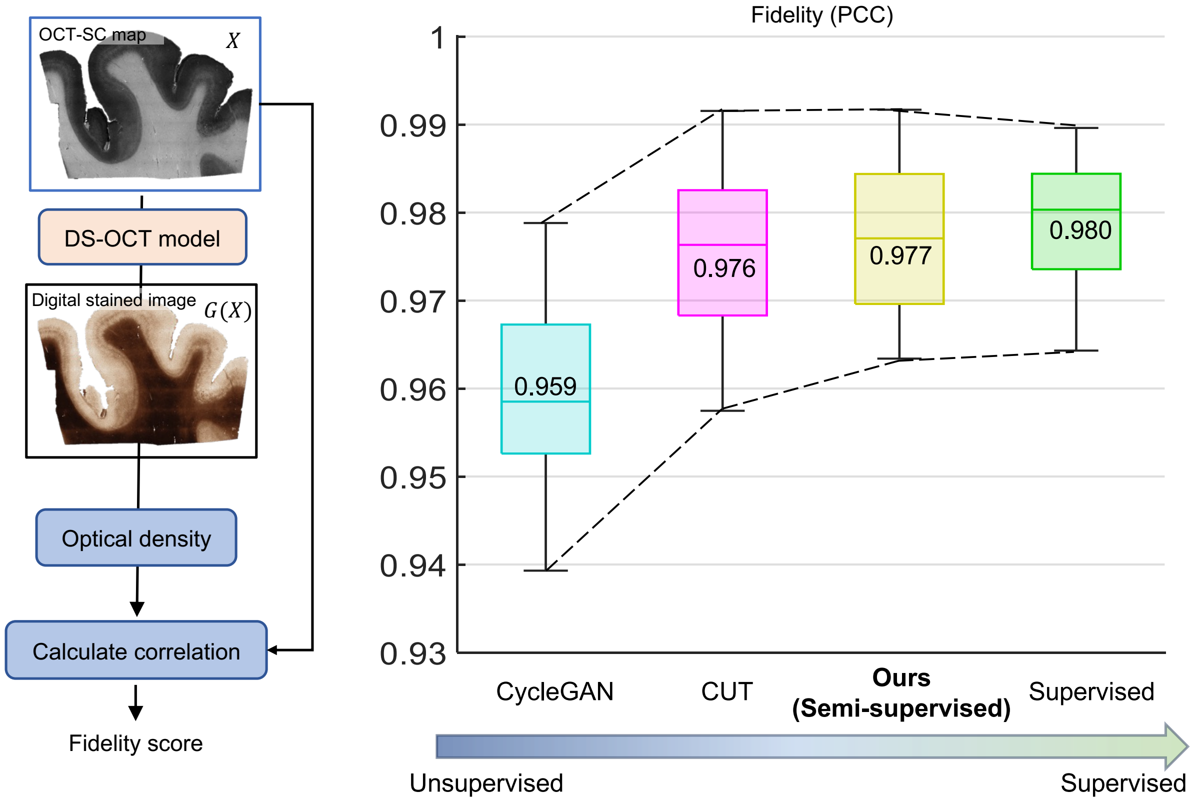

Figure S5. Quantification of hallucinations by DL models on our DS models compared to previous methods. Utilizing well-understood OCT modality as a baseline reference, we evaluate the faithfulness of DL-generated output images using the evaluation pipeline described on the left. We computed fidelity scores of four DS methods: CycleGAN, CUT, our semi-supervised framework, and pseudo-supervised method. Note the pseudo-supervised method is marked as "Supervised" in the figure to save space. With the strength of supervision ranging from low to high, the DL model is expected to generate less hallucinations, which our evaluation results agreed well with.

4. Training details of the deep learning models

In order to ensure a fair comparison, all deep learning models in this study utilize the same ResNet structure[2] as the backbone for the DS generator G. Additionally, uniformity is maintained among the discriminators, which follow the PatchGAN structure, and the multi-layer perceptron (MLP), which adopts a consistent two-hidden-layer structure[3]. For further details regarding the network structures, please refer to Section 10. To identify the optimal model, we conducted empirical optimization of hyperparameters and determined the appropriate number of epochs for early stopping (out of a total of 500 epochs). Our optimization primarily focused on adjusting the loss weights and learning rate through a systematic grid search approach. In terms of the learning rate scheduler, we compared different decay strategies, including linear decay, exponential decay, and cyclic learning rate. After careful evaluation, we found that a linear decay schedule following the first 50 epochs yielded the best results across all models.

As for the first baseline model, we follow the model architecture of the original CycleGAN [2] and utilize a combination of adversarial loss and cycle consistency loss to train two pairs of generators and discriminators (G, D_y) and (F, D_x). To ensure fair comparison, the adversarial loss is the least-squares style GAN loss that is also used in all other models as shown in the main manuscript:

$$L_{\text{GAN}}(D_y) = E_y\left[\left(D_y(y) - 1\right)^2\right] + E_x\left[\left(D_y(G(x)\right)^2\right] \tag{1}$$

$$L_{\text{GAN}}(G) = E_x\left[\left(D_y\left(G(x)\right) - 1\right)^2\right] \tag{2}$$

The cycle consistency loss is formulated as:

$$L_{\text{cyc}}(G) = E_x\left\|F\left(G(x)\right) - x\right\|_1 \tag{3}$$

$$L_{\text{cyc}}(F) = E_y\left\|G\left(F(y)\right) - y\right\|_1 \tag{4}$$

yielding a final full objective of

$$L_{\text{CycleGAN}}\left(G, F, D_x, D_y\right) = L_{\text{GAN}}\left(G, D_y\right) + L_{\text{GAN}}(F, D_x) + \lambda_a L_{\text{cyc}}(G) + \lambda_b L_{\text{cyc}}(F) \tag{5}$$

with two hyperparameters λ_a and λ_b. In our training experiments, we fine-tuned the CycleGAN model and selected the best parameter setting as $\lambda_a = \lambda_b = 10$. The optimized learning rate is 0.0002. We ran 500 epochs of training and used quantitative metrics to monitor the model performance during training. The best model is selected at epoch 465.

For the CUT and FastCUT models, the full objective is a combination of least-squares GAN loss and PatchNCE loss [3] as described in the main manuscript:

$$L_{\text{CUT}}(G, D, f) = L_{\text{GAN}}(G, D) + \lambda_x L_{\text{PatchNCE}}(G, f, x) + \lambda_y L_{\text{PatchNCE}}(G, f, y) \tag{6}$$

As for the CUT model, the PatchNCE loss here is computed both on the input images x and on the output images y with two hyperparameters λ_x and λ_y. We fine-tuned and selected the best parameters as $\lambda_x = 20$, $\lambda_y = 10$. The optimized learning rate is 0.0003. Out of 500 epochs of training, the best model is selected at epoch 190. For the FastCUT model, the PatchNCE loss is only computed on the input images *x* with one hyperparameter λ_x ($\lambda_y = 0$). We fine-tuned and selected the best parameter as $\lambda_x = 20$. The optimized learning rate is 0.0002. The best model is reached at epoch 280.

In our proposed model, the final objective for training G is a combination of adversarial loss, PatchNCE loss, pseudo-supervised loss and registration loss, which are described in the main manuscript:

$$L_{\text{Ours}}(G, D, f) = L_{\text{GAN}}(G, D) + \lambda L_{\text{PatchNCE}}(G, f, x) + \alpha L_{\text{Pseudo}}(G) + \beta L_{\text{reg}}^{II}(G) \quad (7)$$

We fine-tuned and selected the best parameters as $\lambda = 20, \alpha = 10, \beta = 1$. The optimized learning rate is 0.0002. The best model is selected at epoch 260. In addition, when training the model R for registration, we used the following loss function at the pre-training stage:

$$L_{\text{reg}}^{I}(R) = \gamma E_{X,Y} \|X - \phi \circ OD(Y)\|_1 + \eta \|\phi\|_{TV} \quad (8)$$

At the fine-tuning stage, the loss function is:

$$L_{\text{reg}}^{II}(R) = \gamma E_{X,Y} \|G(X) - \phi \circ Y\|_1 + \eta \|\phi\|_{TV} \quad (9)$$

The optimized loss weights for both stages are $\gamma = 100, \eta = 600$ with the learning rate 0.0002.

The DS inference time is the same since all the DS models that have been compared are using the same ResNet-9 blocks backbone. It is important to note that all auxiliary models are used only at the ***training*** phase, ***not*** the ***inference*** phase. The median value of inference time is 13.5s on 3000x4000 pixel WSIs using Nvidia Tesla P100 GPU. We compared the training time of our proposed model with other models as following: CycleGAN: 26.8 hours, CUT: 9.55 hours, FastCUT: 9.48 hours, Ours: 13.6 hours.

5. Quantitative metrics

We first perform pairwise quantitative evaluations of the DS results using traditional image metrics such as mean squared error (MSE), Pearson correlation coefficient (PCC), and color difference (CD). Pairwise comparisons are carried out between whole-slide DS images and registered PS images. Here, for simplicity, we denote the DS modality as X_i^c and PS modality as Y_i^c, where $c \in \{R, G, B\}$ represents different color channels in standard RGB (sRGB) space, and $i \in [1, N]$ indicates pixel indices.

The MSE is calculated as:

$$\mathrm{MSE}(X, Y) = \frac{1}{3N} \sum_{c \in \{R,G,B\}} \sum_{i=1}^{N} (X_i^c - Y_i^c)^2 \tag{10}$$

The PCC is defined as:

$$\mathrm{PCC}(X, Y) = \frac{\sum_{i,c}(X_i^c - \bar{X})(Y_i^c - \bar{Y})}{\sqrt{\sum_{i,c}(X_i^c - \bar{X})^2 (Y_i^c - \bar{Y})^2}} \tag{11}$$

To quantify the color tone similarity in a perceptually uniform color space, we first convert X^c, Y^c from sRGB to CIELAB color space [5], where $c \in \{L, a, b\}$. The CD is then defined as:

$$\mathrm{CD}(X, Y) = \frac{1}{N} \sum_{i=1}^{N} \sqrt{(X_i^L - Y_i^L)^2 + (X_i^a - Y_i^a)^2 + \left(X_i^b - Y_i^b\right)^2} \tag{12}$$

We customize a metric to quantify the similarity of visible cortical layers in DS and PS images, specifically layer IV/V/VI. The layer mask extraction procedure can be found in Section 13. We denote the layer mask (binary image) of DS and PS modality as LX and LY, respectively. The intersection over union score of extracted cortical layer masks (IOU-layers) is defined as:

$$\mathrm{IOU} - \mathrm{layers}\ (X, Y) = \frac{|LX \cap LY|}{|LX \cup LY|} \tag{13}$$

where $|\cdot|$ means the sum of absolute values, $\cap$ and $\cup$ indicates the intersection and union operation respectively.

We further customized unpaired metrics between whole-slide DS images and unregistered PS images. The myelin fiber (MF) segments masks MFX and MFY are extracted as described in Section 13. We compute the length and diameter of individual segments and then calculate the aggregated histograms of the two features as the approximate probability density distributions. The Jensen-Shannon divergence (JS) of MF length distributions p_L and q_L (from DS and PS respectively) is defined as:

$$\mathrm{JS} - \mathrm{MF\ length}\ (X, Y) = \frac{1}{2} D_{\mathrm{KL}}\left(p_L || \frac{1}{2}(p_L + q_L)\right) + \frac{1}{2} D_{\mathrm{KL}}\left(q_L || \frac{1}{2}(p_L + q_L)\right) \tag{14}$$

where

$$D_{\mathrm{KL}}(p||q) = \sum p(x) \log\left(\frac{p(x)}{q(x)}\right) \tag{15}$$

is the asymmetric KL-divergence. Similarly, the JS of MF diameter distributions p_D and q_D is defined by:

$$\mathrm{JS-MF\ diameter}\,(X,Y)=\frac{1}{2}D_{\mathrm{KL}}\left(p_D\,||\,\frac{1}{2}(p_D+q_D)\right)+\frac{1}{2}D_{\mathrm{KL}}\left(q_D\,||\,\frac{1}{2}(p_D+q_D)\right) \tag{16}$$

The MF-segments number is counted as the number of individual segments extracted, where each segment is determined as one connected component (CC) with 2-connectivity.

$$\mathrm{MF-segments\ number}\,(X)=|CC(\mathrm{MFX})| \tag{17}$$

$$\mathrm{MF-segments\ number}\,(Y)=|CC(\mathrm{MFY})| \tag{18}$$

The MF-area percentage is calculated by dividing the area of MF mask (MFX, MFY) by the area of sample mask (SX, SY), which is described in Section 13:

$$\mathrm{MF-area\ percentage}\,(X)=\frac{|\mathrm{MFX}|}{|\mathrm{SX}|} \tag{19}$$

$$\mathrm{MF-area\ percentage}\,(Y)=\frac{|\mathrm{MFY}|}{|\mathrm{SY}|} \tag{20}$$

6. Additional visualization results of DS results

Figure S6 provides additional examples of our results obtained from cortex tissue section samples. The figure includes four WSIs including OCT-SC, DS, and PS images (adjacent sections) arranged from left to right. Within each sample, we have selected two smaller region-of-interest (ROI) areas to highlight finer details. Notably, the color contrasts remain consistent, and the preservation of vessel structures is evident in our DS result, indicated by small white blobs. It is worth noting that the cortical layer separation appears to be more pronounced in the DS images compared to the PS modality, primarily due to reduced staining variability.

The first row of the figure displays one of the relatively ideally stained samples. However, in the remaining samples, we observe instances of under-staining, over-staining, or non-uniform staining patterns in the PS images. Overall, we observe comparable visualization of fine features and superior staining consistency across samples and ROIs in the DS images compared to the PS images. These additional examples align with the results presented in the main manuscript, further supporting our findings.

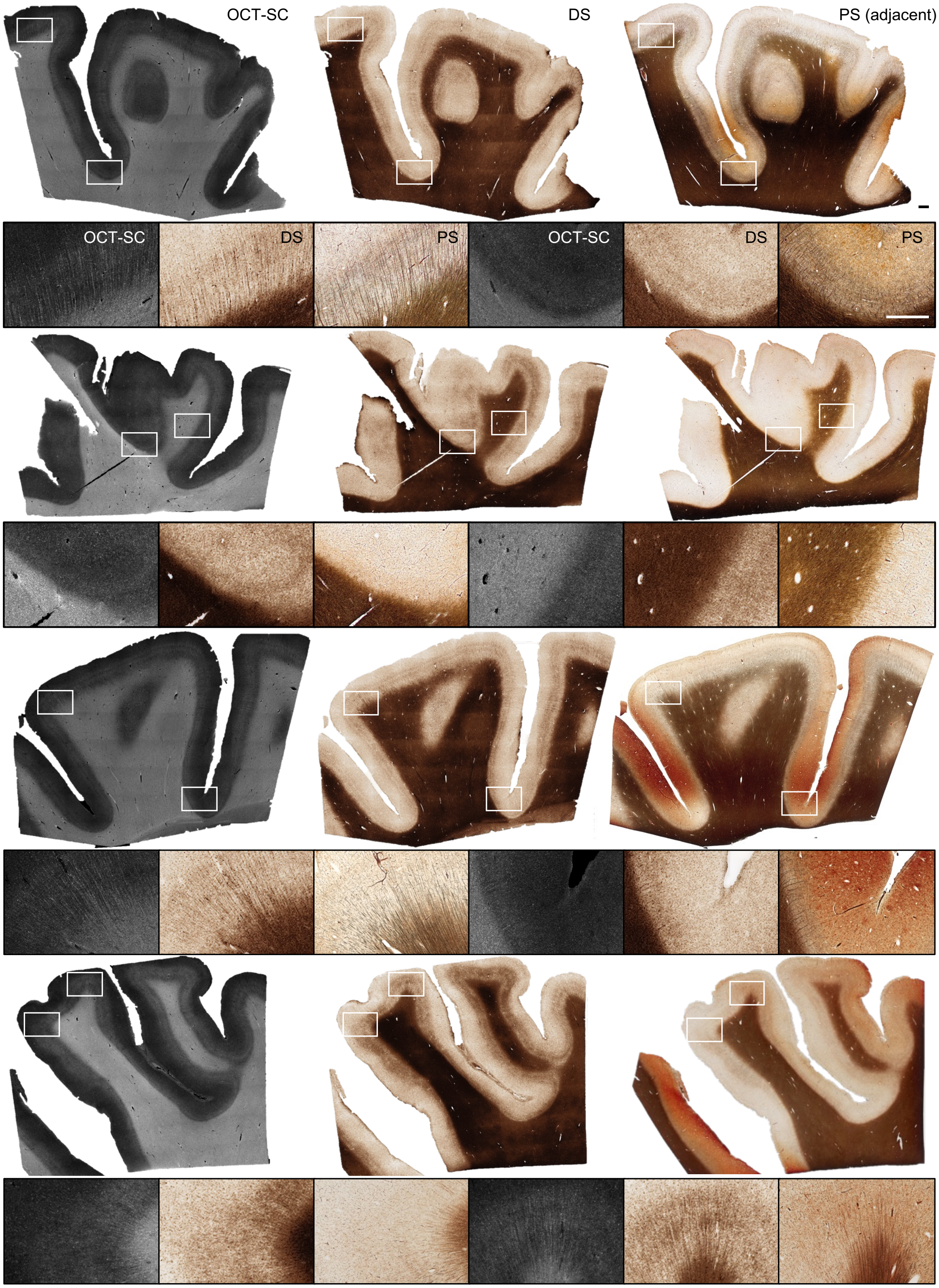

Figure S6. Additional visualization of DS results. Cases include Row 1: ideal staining; Row 2: under-staining sample; Row 3: over-staining sample; Row 4: non-uniform staining. Scale bars are 1 mm.

7. Additional examples of improved layer differentiation in DS results

Figure S7 provides additional examples highlighting the improved differentiation of cortical layers in our DS results from cortex tissue section samples. The figure includes four examples of WSIs of DS and PS images (adjacent sections). Within each sample, two smaller ROIs are selected. To enhance the contrast of cortical layers, the same image enhancement process, utilizing contrast-limited adaptive histogram equalization (CLAHE) [6] and conversion to grayscale, is applied to each ROI.

Across all the examples, we consistently observe distinct double band features present in the zoomed-in ROIs of the DS results, whereas only a portion of these features is visible in the PS results. Moreover, within the same sample, the DS images exhibit uniform double band features in both sulcus and gyrus regions, whereas the PS images only exhibit some of these features. It is important to note that the PS modality demonstrates instances of both over-staining and under-staining, leading to inferior visualization of cortical layers. Consequently, the DS images facilitate easier differentiation of cortical layers compared to the PS modality due to reduced staining variability.

These additional examples serve as further evidence supporting our main manuscript's argument that DS reduces staining variability, enabling improved differentiation of cortical layers.

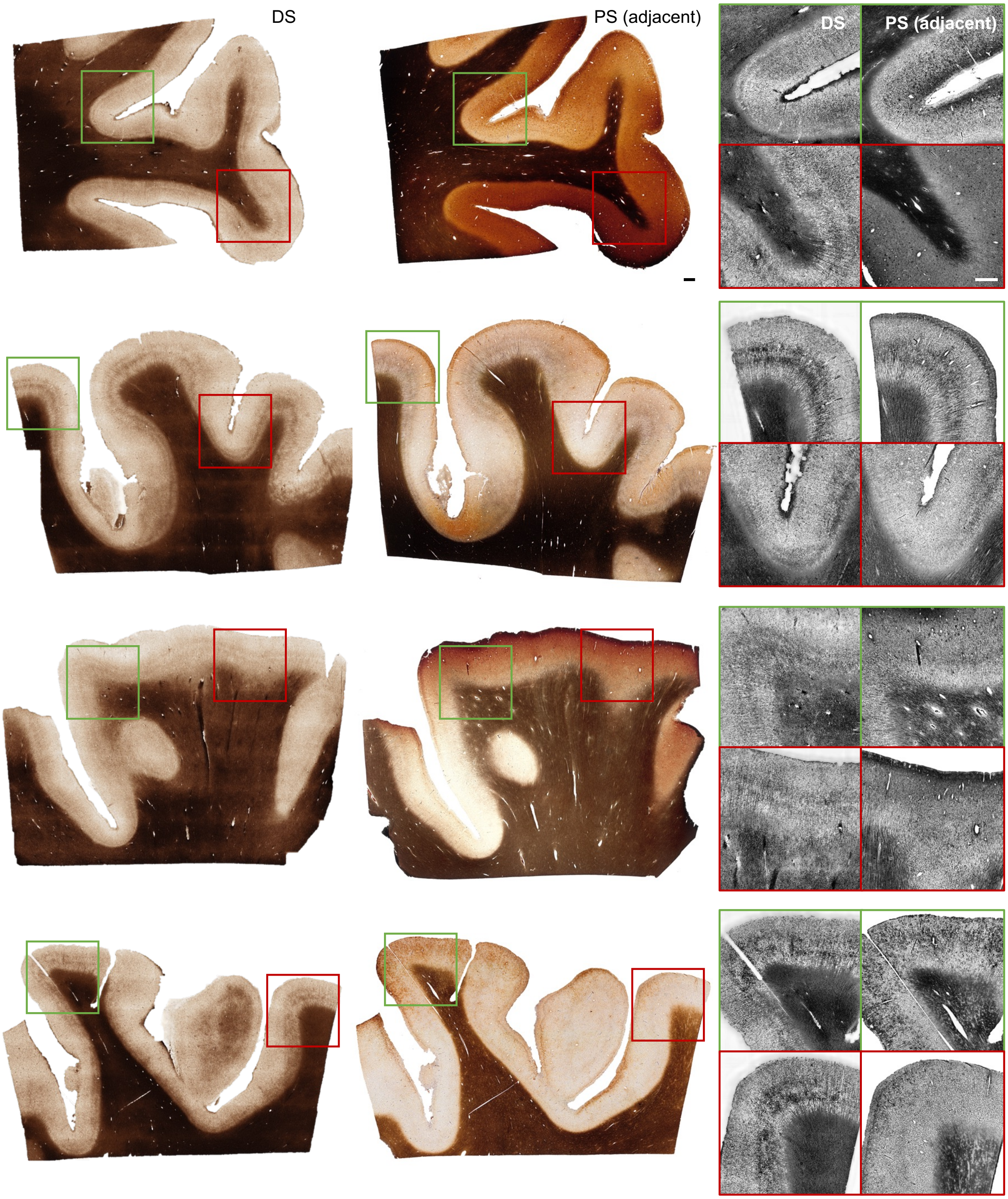

Figure S7. Additional examples of improved layer differentiation in DS results. The DS and PS WSIs from four additional cortex tissue samples are shown on the left panel. Contrast enhanced grayscale images are shown in zoom-in ROIs. For these ROIs, The DS and PS ROIs are shown in order from left to right, while different ROIs are shown from top to bottom and marked by green and red boxes. Scale bars are 1 mm.

8. Additional examples of DS model generalization performance

In Figure S8, we provide additional examples that illustrate the generalization capabilities of our DS model to other anatomical regions and OCT imaging setups. The figure showcases four distinct examples, each displaying OCT-SC, DS, and PS images of adjacent sections, acquired under different experimental conditions and S-OCT setups that differ from those presented in the main manuscript.

In Figure S8A, we present the results obtained from a cerebellum sample using three modalities. Due to the presence of significant speckle noise in the OCT-SC image, a preprocessing step involving outlier removal and strong averaging was applied, resulting in an over-smooth appearance. Consequently, our DS result also exhibits smoothing artifacts. Despite the reduced resolution, we observe similarities in color and layer contrast between DS and PS images, which differ noticeably from samples obtained from the cortex region. Within the sample, we identify three distinct layers stained with light-brown, dark-brown, and black, progressing from the outer to inner regions. Manual annotation of corresponding regions of interest (ROIs) is represented by two red boxes, highlighting the distortion in sample geometry. For the remaining samples with less distortion, coarsely registered ROIs are displayed side by side.

Figure S8 B, C, and D depict three samples from different anatomical brain regions: Somatosensory, BA21, and SupFrontal, respectively. The OCT-SC modality demonstrates some degree of contrast variability due to system imperfections and strong stitching artifacts. Consequently, the DS results also exhibit stitching artifacts and less staining uniformity. Given the presence of these artifacts, visualizing myelin fibers or fine features in the DS results becomes challenging. However, coarse cortical layers can still be observed in the DS images as evidenced by dark ridges. Within the gray matter region, DS successfully differentiates two cortical layers in Figure S8 B and D, and a single cortical layer in Figure S8 C, aligning well with the PS results.

In summary, it is evident that the performance of our current DS-OCT model is influenced by speckle noise and stitching artifacts. These results highlight the dependence of our DS-OCT model on the imaging quality of the S-OCT setup and data processing pipeline.

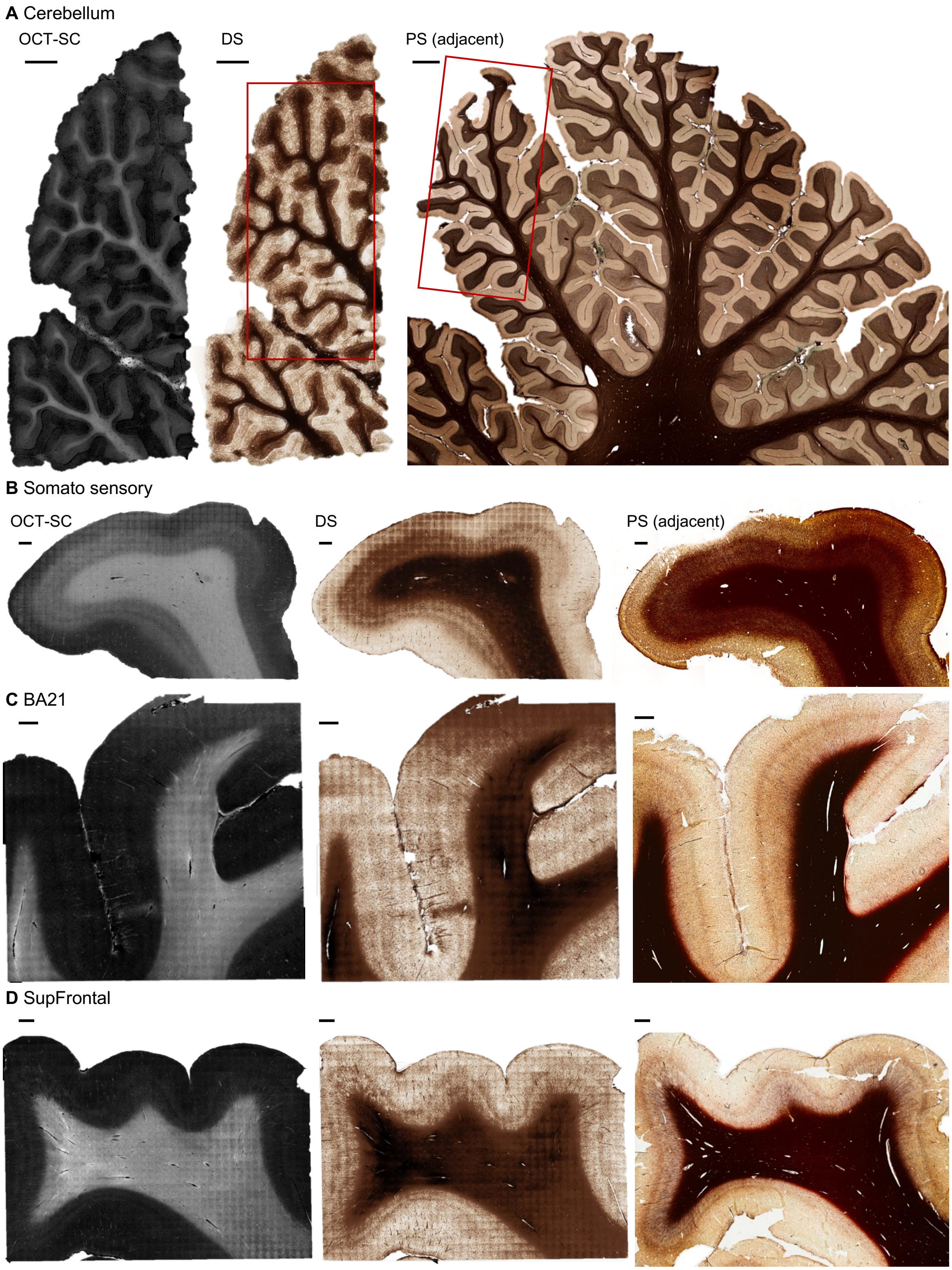

Figure S8. Additional examples of DS-OCT generalization performance. Four additional examples of OCT-SC, DS and PS images (of adjacent sections) are shown. (A-D) tissue sections from four anatomical regions: cerebellum, somatosensory, BA21, and SupFrontal, respectively. In (A), red boxes are coarsely registered ROIs. Scale bars are 1 mm.

9. Image processing pipeline

As described in the "Image processing" of the main manuscript, Figure S9 serves to further elucidate the image processing pipeline employed for OCT-SC, DS, and PS images. Each subfigure provides a visual representation of the specific preprocessing steps applied to the respective image modality.

In Figure S9A, we present the preprocessing steps implemented for PS images. This involves background removal, intensity normalization, and color transfer techniques to enhance the visual quality and ensure consistency across images.

Figure S9B focuses on the preprocessing steps tailored for OCT-SC images. The aim here is to mitigate the background interference and correct the over-smooth values typically observed around vessel region edges. These processing steps contribute to improving the overall clarity and accuracy of the OCT-SC images.

Finally, Figure S9C showcases the post-processing steps carried out on DS images. This includes sample masking and the application of white matter (WM) masking. By employing these techniques, we are able to isolate and highlight specific areas of interest within the DS images, facilitating a more detailed analysis.

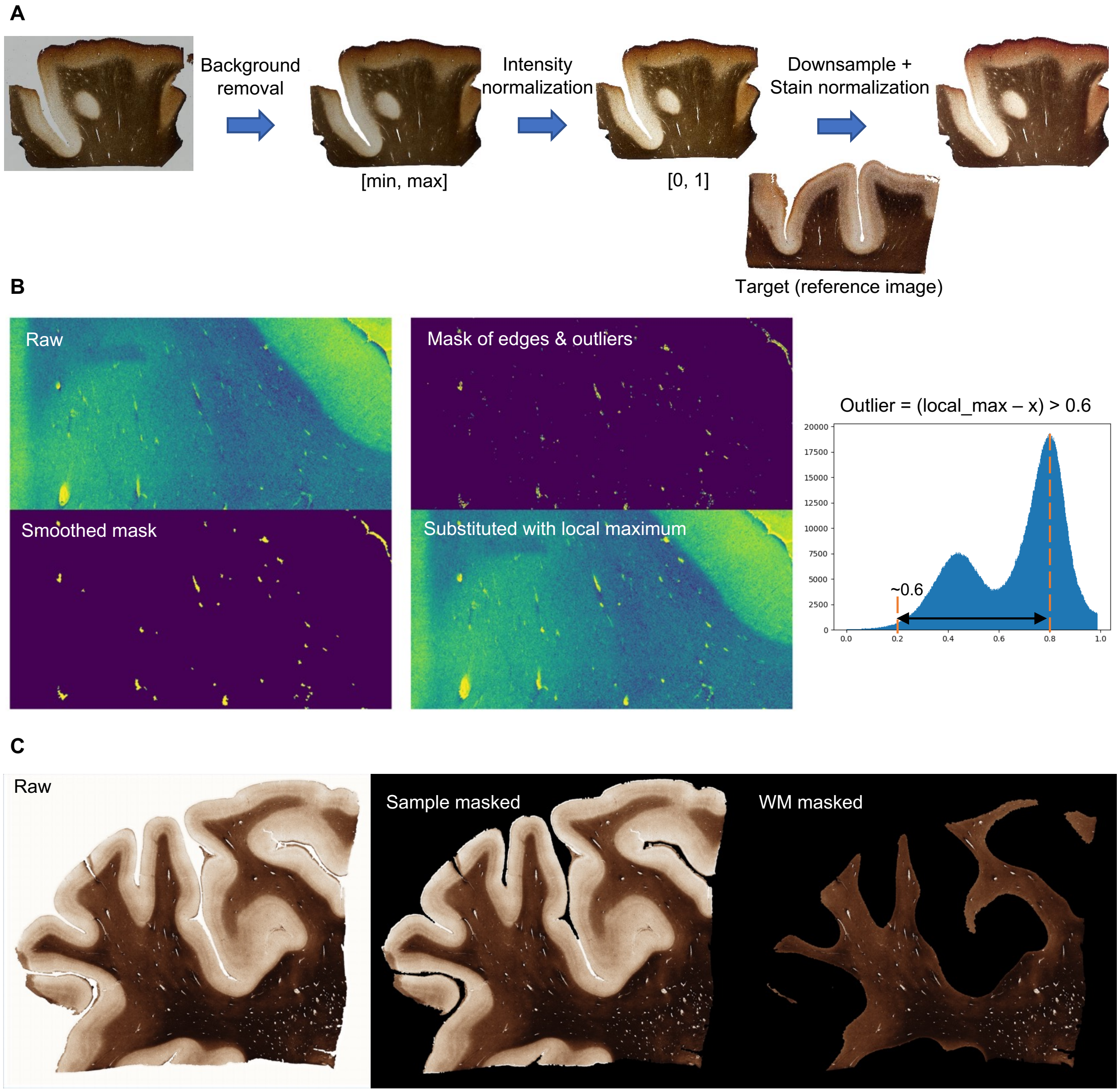

Figure S9. Image processing pipeline. (A) Preprocessing of PS images includes background removal, intensity normalization, resolution matching (downsampling) and color transfer. (B) Preprocessing of OCT-SC images include edges & outliers masking, mask smoothing and maximum filtering. The outlier mask is determined by comparing pixel values to the local maximum shown on the right panel. (C) Post-processing of DS images. The second and third column shows results with sample masked and white matter (WM) region masked.

10. Additional details of our semi-supervised deep learning framework

As outlined in the 'Semi-supervised deep learning framework' section of the main manuscript, Figure S10 and Figure S11 provide additional insights into the training details of our approach. The figures illustrate various aspects of the training process.

In Figure S10A, we demonstrate the semi-supervised training of the DS generator G, which combines contrastive learning, adversarial learning, pseudo-supervised learning, and learnable registration. The OCT-SC map X and the corresponding PS image Y from an adjacent slice are utilized during training, but no exact supervised pairs of X and Y are explicitly provided to G. The training process involves alternate optimization of G and the learnable registration component R at different image scales. R is held fixed while optimizing G, and the updated G provides informative supervision for the subsequent iteration of R. Patch-wise losses are formulated and combined into a total loss during the training of G.

In Figure S10B, we illustrate the training of unsupervised cross-modality image registration. WSI losses are formulated between different pairs of image modalities at two distinct training stages. See Figure S11 for more details.

In Figure S11A, we illustrate the two-stage training approach used. We combine the loss from two modalities to train the registration module to improve accuracy. The deformation is generated by the registration network in the same way, but two loss terms are formulated on different outputs. At the pre-training stage, R is trained independently across the entire WSI dataset. The formulation of L_{reg}^{I} as detailed in Section 4 is an unsupervised loss utilizing a similarity prior between X and $OD(Y)$. During this stage, the focus is on establishing a fast-trained coarse registration model. Notably, the first-stage loss is independent of G thus can be pre-trained faster and at WSI scale to reduce training cost. At the fine-tuning stage, R and G are alternately trained using selected high-quality WSIs (training data of G). The second-stage loss L_{reg}^{II} (see Section 4) is defined in the stained modality as the difference between the DS image and the deformed PS image. During the fine-tuning, the loss is dependent on both the DS net and the registration net, which encourages collaborative learning. This enables the refinement of the registration model as well as weakly-supervised learning of the DS model.

In Figure S11B, we explain the alternate training strategy in the fine-tuning stage. At the second stage, the loss L_{reg}^{II} for the DS network G needs to be at patch scale to be incorporated with previous patchwise contrastive loss. However, the registration network R needs to be trained at WSI scale to account for the global geometry correction. As a result, we alternately train the DS and registration model at different image scales to enable collaborative training between them. Essentially, the unsupervised cross-modality image registration network helps the DS model to learn a better local color tone mapping from unaligned images, even though it cannot accurately register images with pixel alignment.

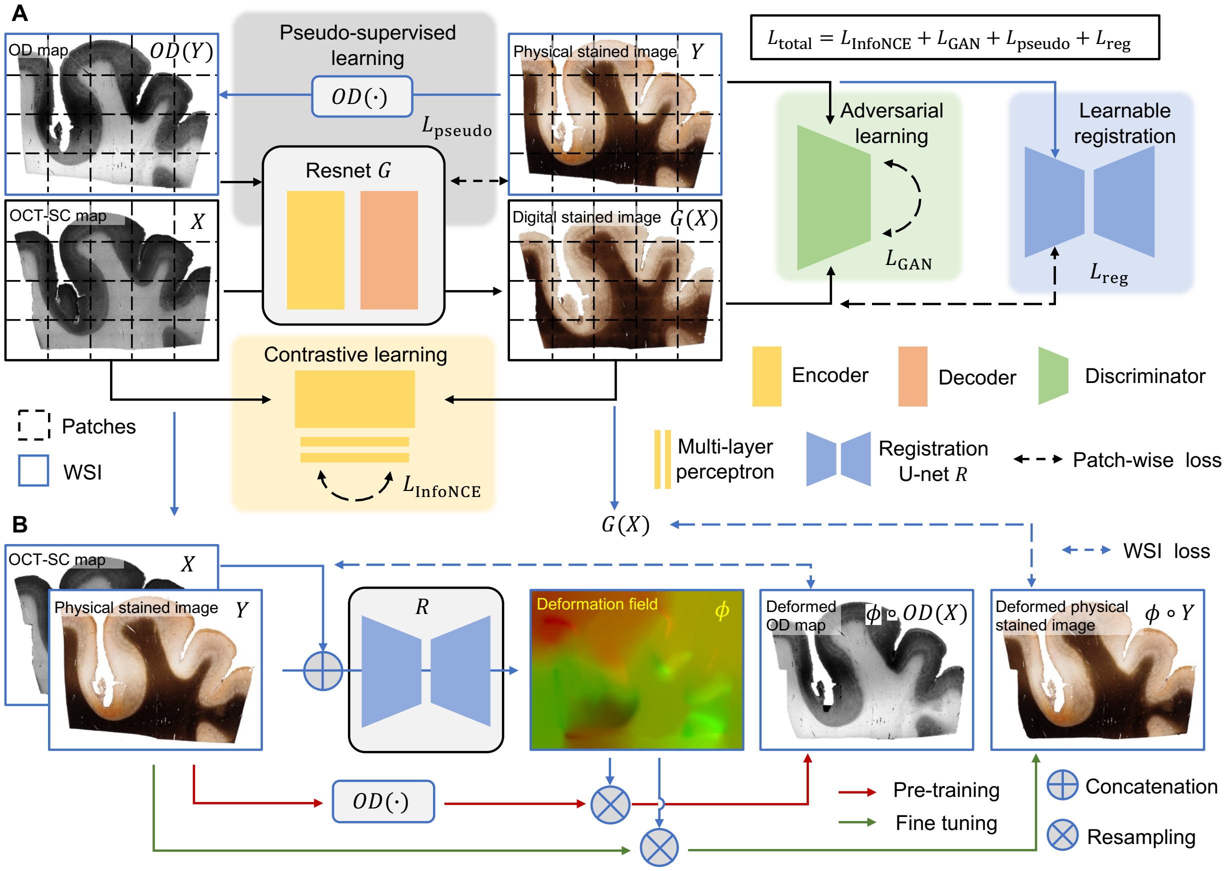

Figure S10. Detailed semi-supervised training framework of our DS model. (A) Semi-supervised training of DS generator G combining contrastive learning, adversarial learning, pseudo-supervised learning and learnable registration. Patch-wise losses are formulated and combined in the total loss when training G. Blue arrows and blue boxes indicate images are processed at the WSI scale. Black arrows, dotted black double-arrows and dotted black boxes indicate images are in patch-wise scale and are processed patch-wise. (B) Unsupervised cross-modality image registration. WSI losses are formulated between different image-modality pairs at two training stages. R is trained stand-alone at the pre-training stage, where similarity prior of X and $OD(Y)$ is used. At the fine-tuning stage, R is trained collaboratively with G using alternate optimization. Red arrows indicate the forwards pass during pre-training, and green arrows indicate the forward pass during fine-tuning. All images are processed as WSI scale in (B).

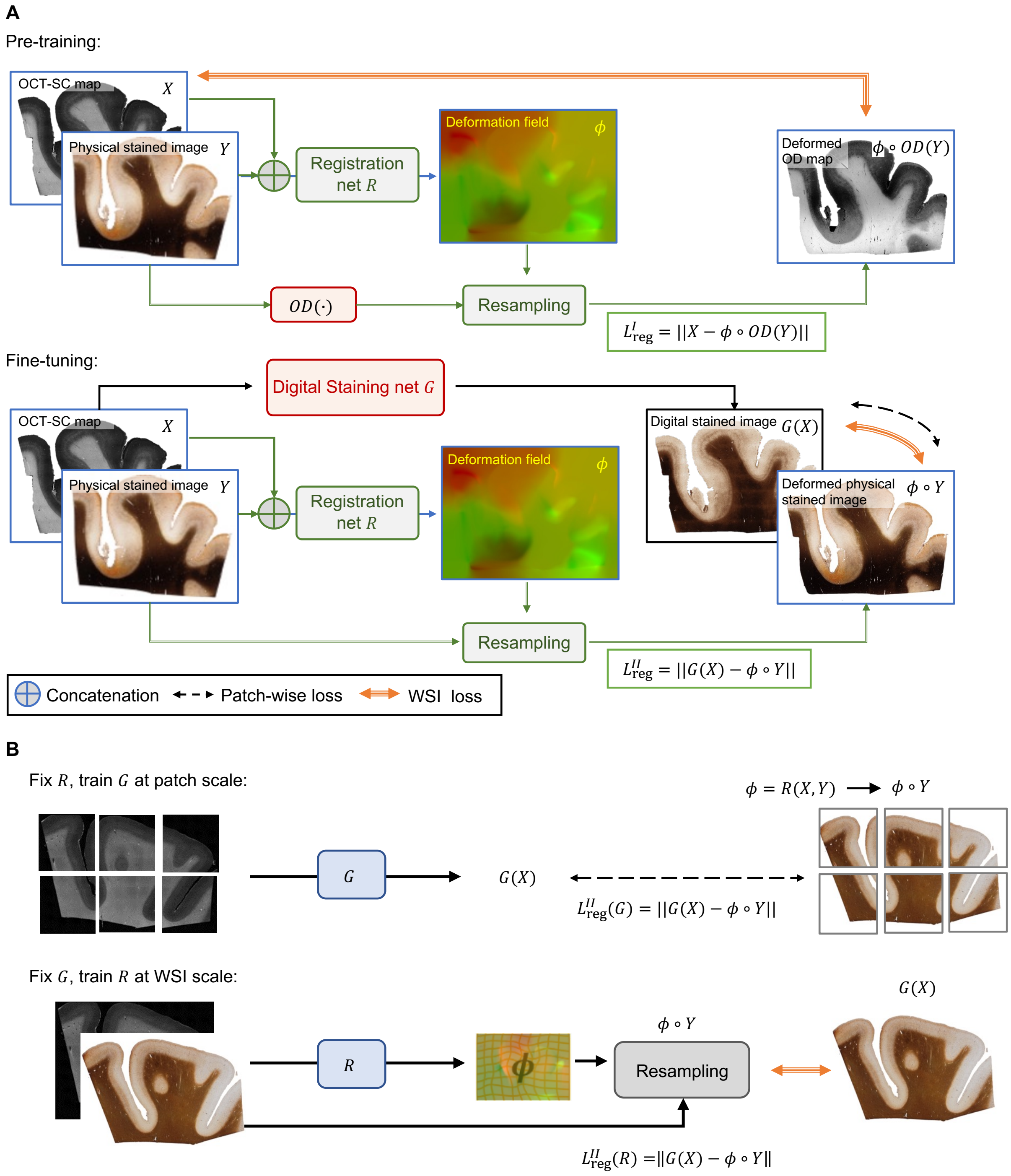

Figure S11. Two-stage, alternate training with different image scales. (A) The deformation field is the output of registration network R given a pair of unaligned images X and Y. At the pre-training stage, it is then used to resample the OD map and compute the difference between SC image and deformed OD image. At the fine-tuning stage, the PS image Y is deformed to compute the difference with the DS image. The collaborative training of G and R will minimize this loss using alternate optimization updates. (B) We train the registration model at the WSI scale and the DS model at the image patch scale. At each alternate step, one of the networks is fixed and the other is updated by backpropagating the loss gradients at different image scales.

11. Deep Learning model architectures

The network structures of each component model are depicted in Figure S12, providing a comprehensive overview of the architecture employed in each model. The generator G follows the ResNet architecture with 9 ResBlocks [2]. The registration net R is a ResU-net with 6 downsampling blocks. The discriminator follows the structure of 70 × 70 PatchGAN [2]. The MLP used in contrastive loss is a simple two-layer fully connected network with 256 hidden neurons in each layer [3].

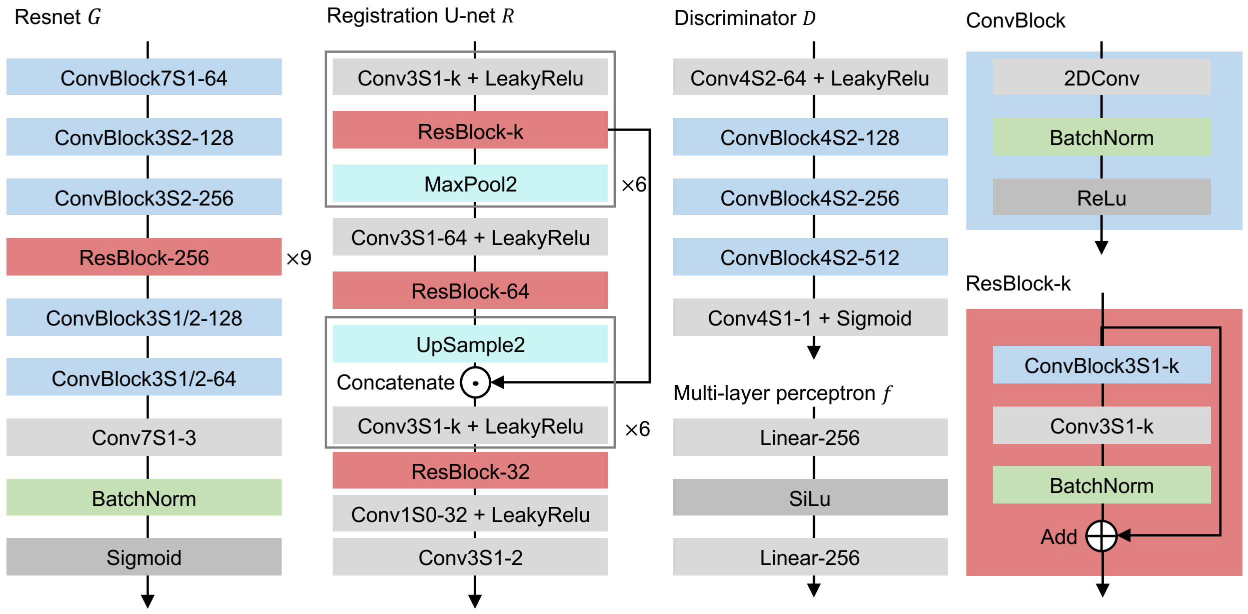

Figure S12. Model architecture details. The building blocks are convolutional blocks (ConvBlock) and residual blocks (ResBlock). The naming rules are as follows: Conv(i)S(j)-(k) represents an ii 2D convolution with k filters and stride j, where fractional stride size indicates transpose convolution; ConvBlock(i)S(j)-(k) represents a block consisting of an ii 2D convolution with k filters and stride j, followed by a batch normalization (BatchNorm) layer and a ReLu activation. ResBlock-(k) represents a ConvBlock3S1-k followed by a Conv3S1-k and BatchNorm; Linear-k is a fully connected layer with k hidden neurons. All ReLus in ConvBlocks of discriminator D are leaky ReLu. We use all leaky ReLus with a slope of 0.2.

12. Ablation study of the modules in our proposed semi-supervised method

Our proposed method consists of three major components: CUT, registration learning, pseudo-supervised learning. Our full model is compared to the model trained only on (1) CUT + registration learning module, (2) registration module only, (3) CUT + pseudo-supervised learning module, (4) pseudo-supervised learning module. Models in each ablation studies are fine-tuned. Our full training framework provides consistently better performance both visually and quantitatively, as shown in Figure S13.

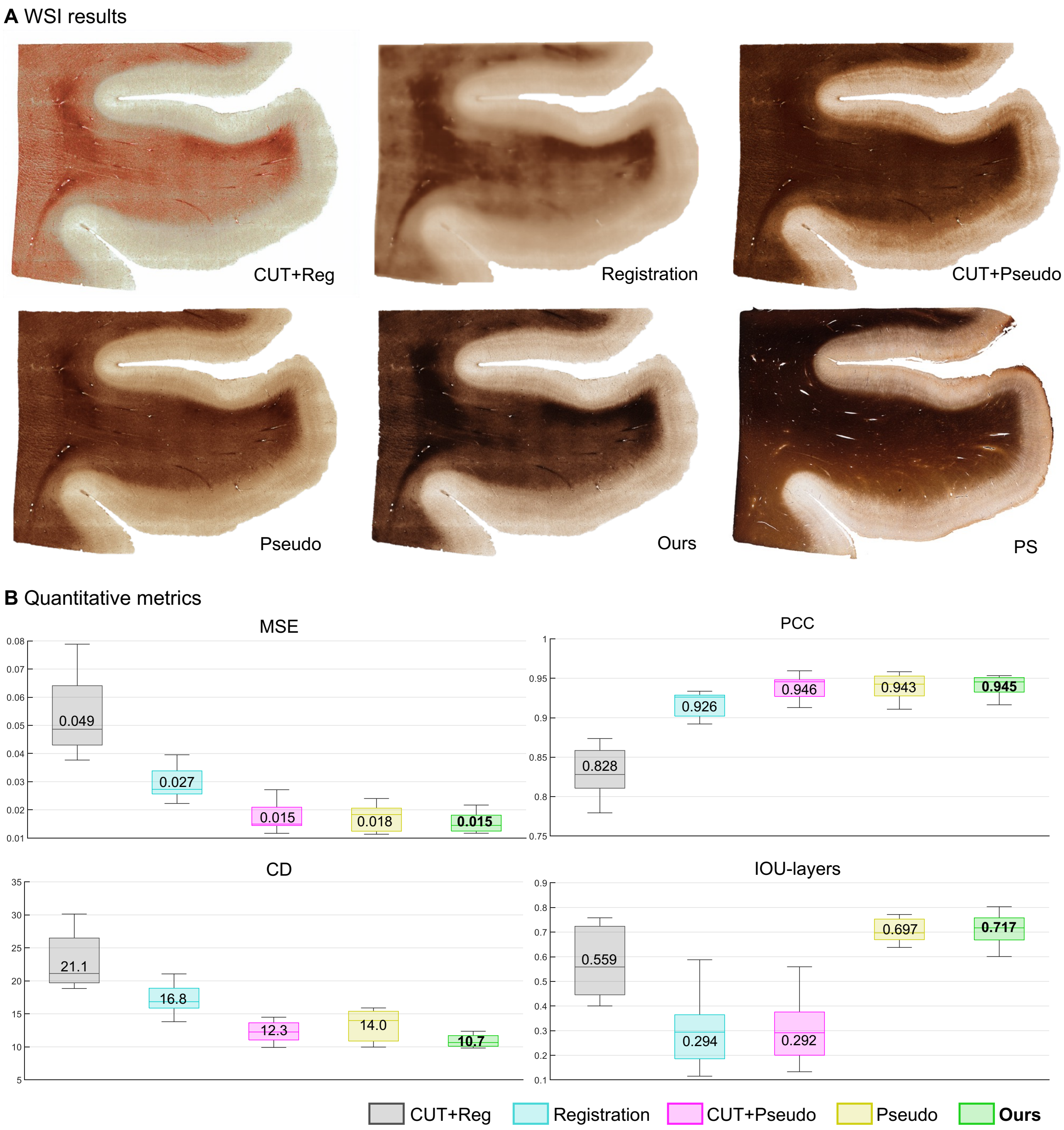

Figure S13. Ablation study of our proposed semi-supervised methods. (A) Visualization example of WSI results. (B) Pair-wise quantitative metrics comparisons. Our proposed method consists of three major components: CUT, registration learning, pseudo-supervised learning. Our full model is compared to the model trained only on (1) CUT + registration learning module, (2) registration module only, (3) CUT + pseudo-supervised learning module, (4) pseudo-supervised learning module. Models in each ablation studies are fine-tuned. Our full training framework provides consistently better performance both visually and quantitatively.

13. Image analysis for fiber segmentation and quantification

In Figure S14, we present the image analysis pipeline employed for myelin fiber extraction and quantification. The figure provides a step-by-step illustration of the process involved.

Figure S14A focuses on the segmentation of cortical layers IV/V/VI. Initially, the DS and PS images are converted to grayscale, and the initial sample mask is generated by applying thresholding using the triangle's method [7]. The sample mask is then refined through the removal of small objects and small holes using a structure element of size 256 and 512, respectively. Erosion is subsequently performed with a square kernel of size 75. To enhance the efficiency of this operator with large kernel size, as an alternative approach, we employ a Euclidean distance transform on the binary sample mask image and apply thresholding with a threshold value of 75.

The next step involves the segmentation of a GM mask to exclude the white matter WM region. This is achieved through the minimum thresholding method, followed by the filling of white holes and dilation using a square kernel of size 8. To further refine the segmentation, a brown color mask is extracted to eliminate any remaining regions that are not stained brown. A squared error map is computed by subtracting the mean value of the WM region from the original image and squaring the residual. Thresholding is then performed on this map using the mean method, followed by the removal of small holes and erosion. Finally, the three masks (sample mask, gray matter mask, and brown color mask) are combined through pointwise multiplication, resulting in an estimation of the mask representing the layer IV/V/VI regions. These layer masks are utilized for quantitative evaluation in Section 3.

In Figure S14B, the Frangi filter followed by a thresholding operation is applied to extract tubeness structures, which, in our case, predominantly correspond to myelin fibers. The Frangi filtered mask and the extracted layer mask are combined to segment the fiber tract mask within the layer IV/V/VI region. Subsequently, this mask is utilized to estimate the length and diameter of each individual fiber segment using the scikit-image property measure [7]. The length and diameter histograms are compared between the DS and PS images, and further analysis such as the computation of Jensen-Shannon divergence can be performed to assess the differences between the two distributions. Importantly, this analysis is carried out on population-level features, not necessarily on registered images, enabling unpaired quantitative evaluation in Section 3.

We note that OCT-SC and our DS technique can visualize myelinated axon bundles that consist of multiple axons, at ~12μm resolution, but cannot resolve finer structures due to physical resolution limit and speckle noise in the raw OCT data. This point is further illustrated in Figure S15.

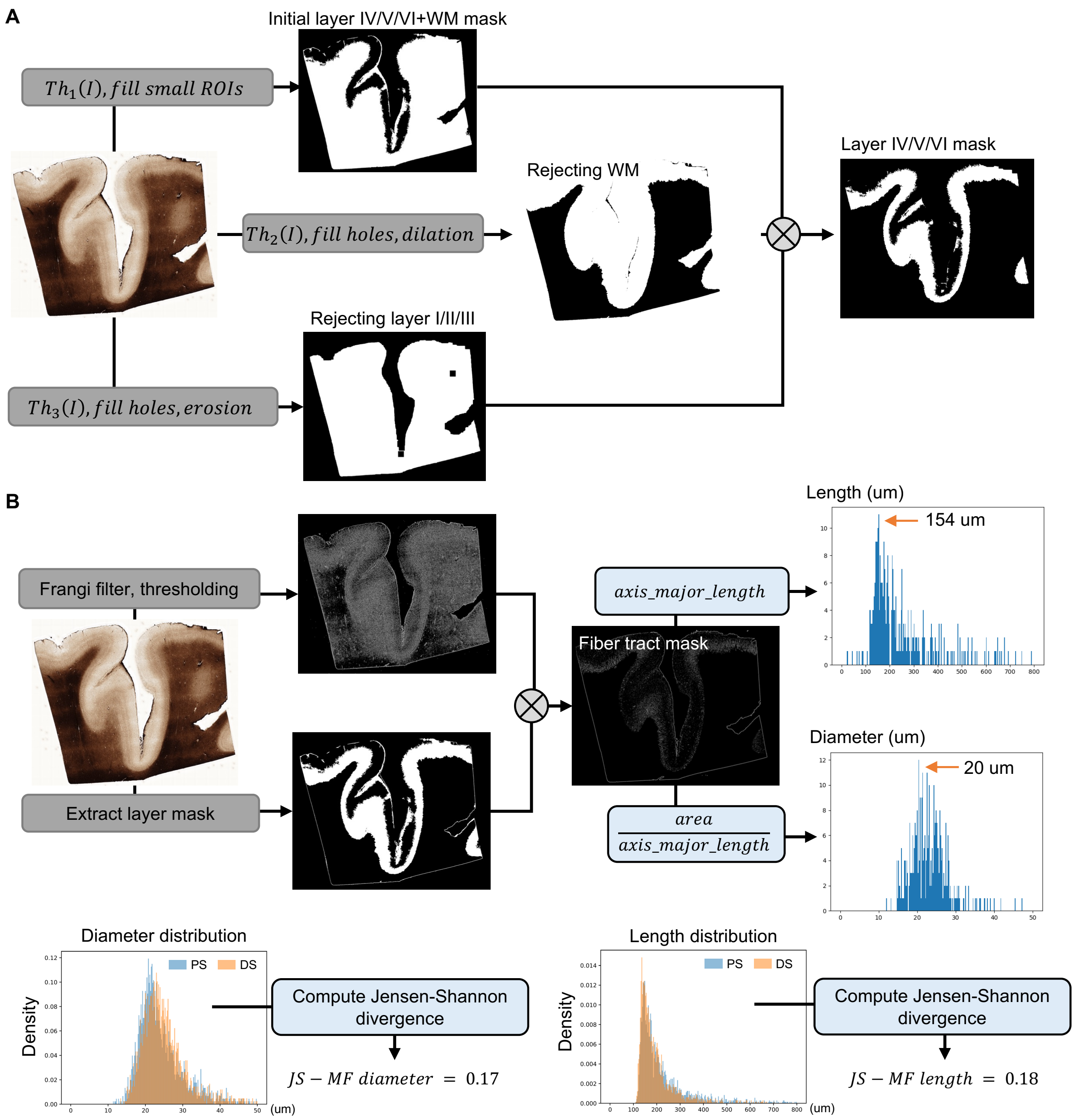

Figure S14. Image analysis for fiber segmentation and quantification. (A) The mask of cortical layer IV/V/VI is segmented by combining an initial layer + white matter (WM) mask, a gray matter (GM) mask and brown color mask. The layer masks are directly used for quantitative evaluation. (B) Frangi filter and layer mask are combined to segment fiber tracts within layer IV/V/VI. Myelin fiber features such as lengths and diameters are extracted to compute histograms. Further quantitative metrics such as Jensen-Shannon divergence are computed from two histograms of extracted features.

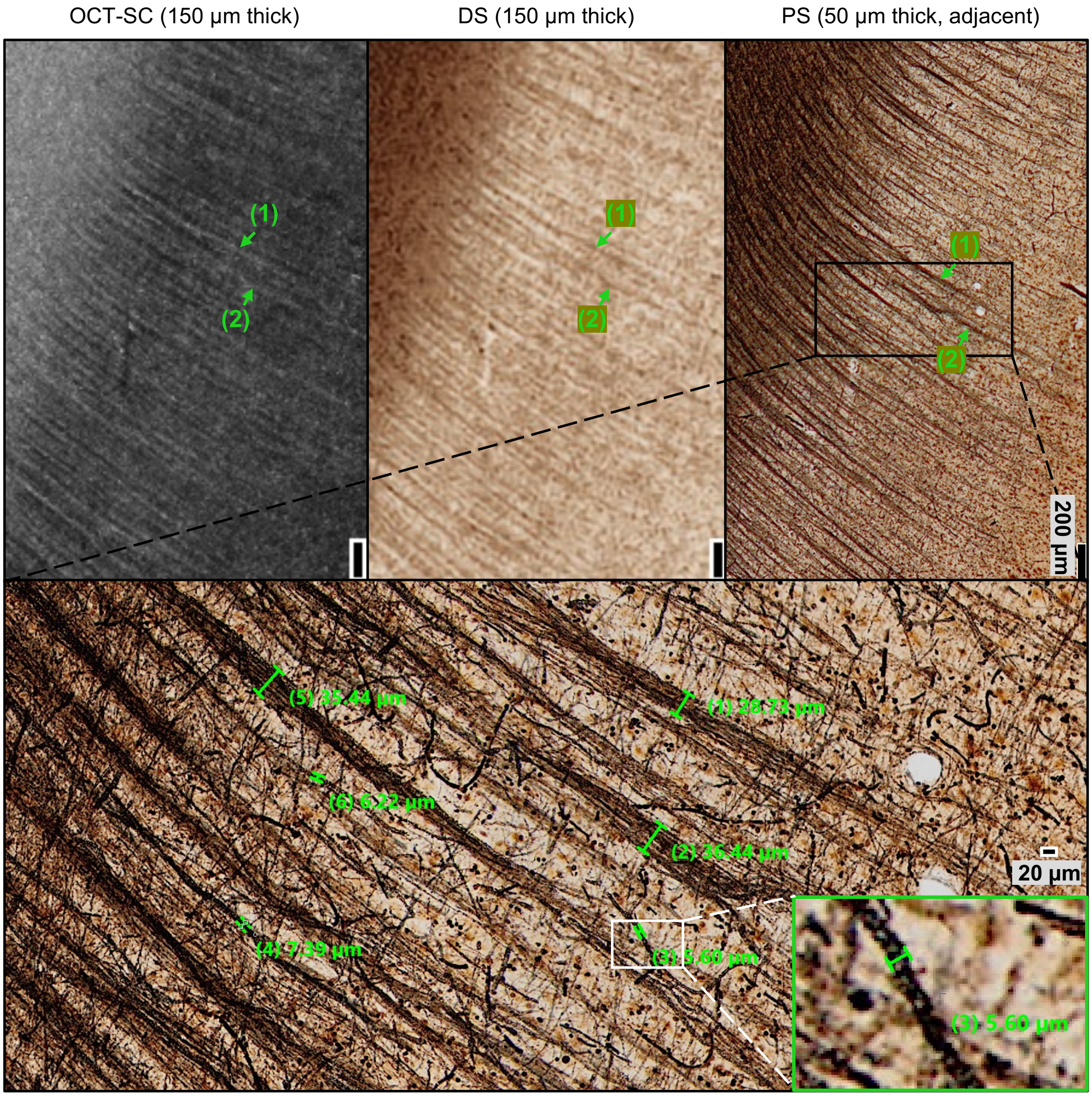

Figure S15. Visual comparisons of ~20 µm myelin fiber features in our OCT-SC, DS-OCT images as well as high-resolution brightfield physically stained images. On the top, myelinated axon bundles (with ~20 µm resolved features) are cross-annotated and shown in (1) and (2) with green arrows. Note the DS image shows no visible hallucination as compared to OCT-SC image. Slight inconsistencies between DS and PS images are due to slice content mismatch (the OCT-SC and DS are both imaging 150 µm thick slice while PS is 50 µm thick adjacent slice). On the bottom zoomed-in FOV, those axon bundles are clearly visualized as multiple myelinated axons grouped in (1), (2) and (5), with 20~40 um diameters. Individual myelinated axons are annotated as (3), (4) and (6) with sub-10um diameters. On even higher zoomed-in view of (3), the axon diameter is clearly labeled and measured as 5.6 um.

14. Color-intensity correlation analysis

Our DS-model does not learn a trivial intensity-to-color mapping. To support this argument, we perform color-intensity correlation analysis and compare our DL-based DS method to a trivial pixel inverse mapping method in Figure S16.

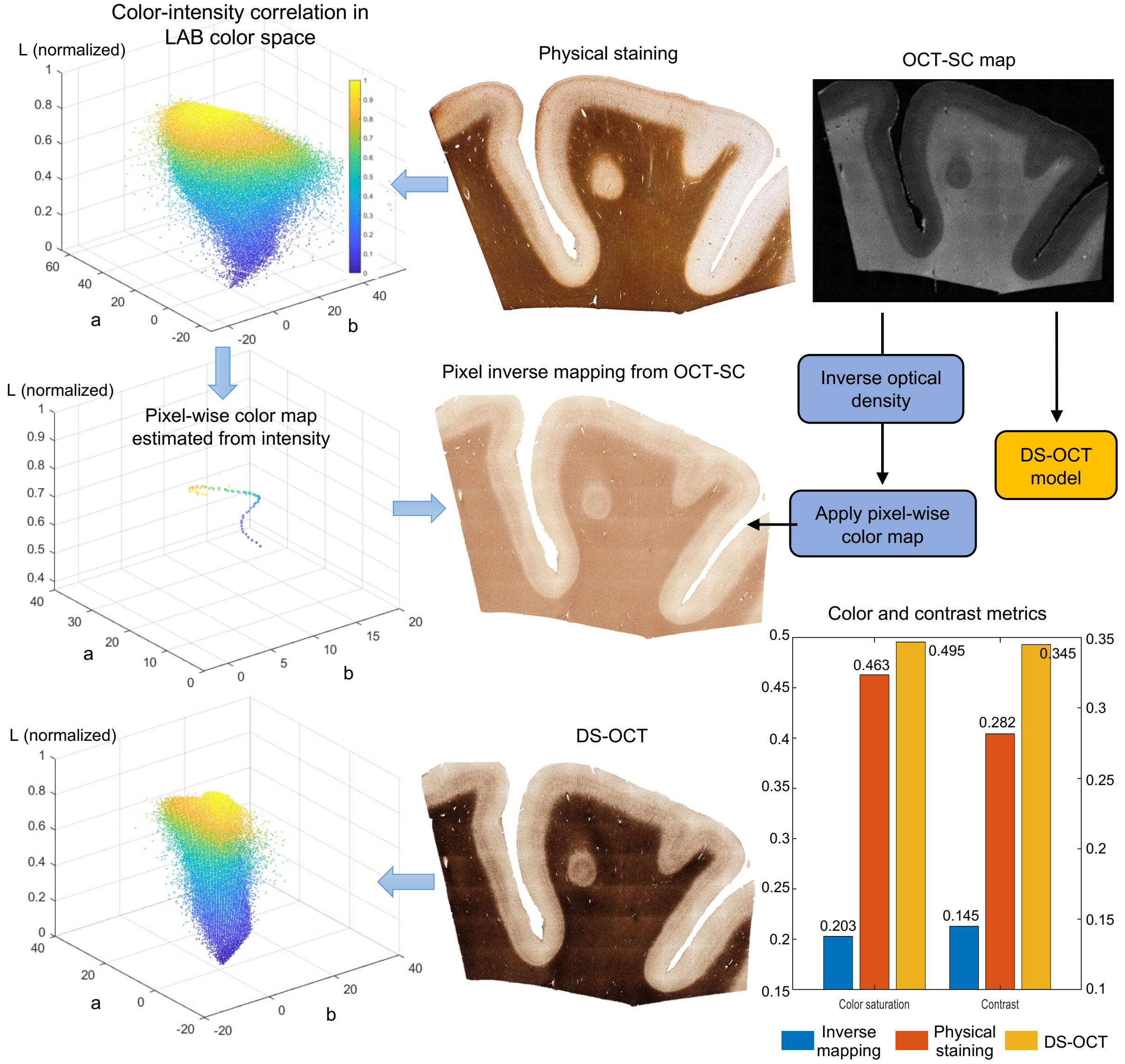

Figure S16. Color-intensity correlation analysis and comparison of our DL-based DS method to pixel inverse mapping method. We show scatter plot of the pixel color (a, b) and intensity (L) values in the perceptually uniform LAB color space to visualize the color-intensity correlation. It is evident there is no one-to-one correlation between (a, b) and L. The simplified inverse color map is estimated by mapping each intensity value L to the corresponding average color component (a, b). This color map can be applied to OCT-SC images after the inverse optical density conversion. The inverse mapping results compared to our results showed significantly lower local color saturation and global contrast.

15. Quantification of volumetric geometry distortion

In our main manuscript, we showcase volumetric staining on cubic centimeter-scale brain tissue enabled by our technique that combines S-OCT and DS. Our technique significantly reduces tissue distortion and misalignment during the 3D reconstruction process suffered by the traditional 3D pathology technique. We demonstrate 3D DS on a 4 cm × 5 cm × 1.2 cm brain tissue block that was not used for training our DS model. We show that our method can preserve the intricate 3D brain structures in both gray matter and white matter regions. Moreover, we visualize the 3D vessel network in the white matter.

To quantify volumetric geometry distortion, we compute the Pearson correlation coefficients (PCC) on adjacent slice pairs in DS and PS results. Using PCC as a geometry distortion metric, our DS-OCT showed drastic improvement for distortion-free 3D imaging. Meanwhile, the PCC score of DS in white matter region indicates smooth transition of white matter-gray matter boundaries. This is shown in Figure S17.

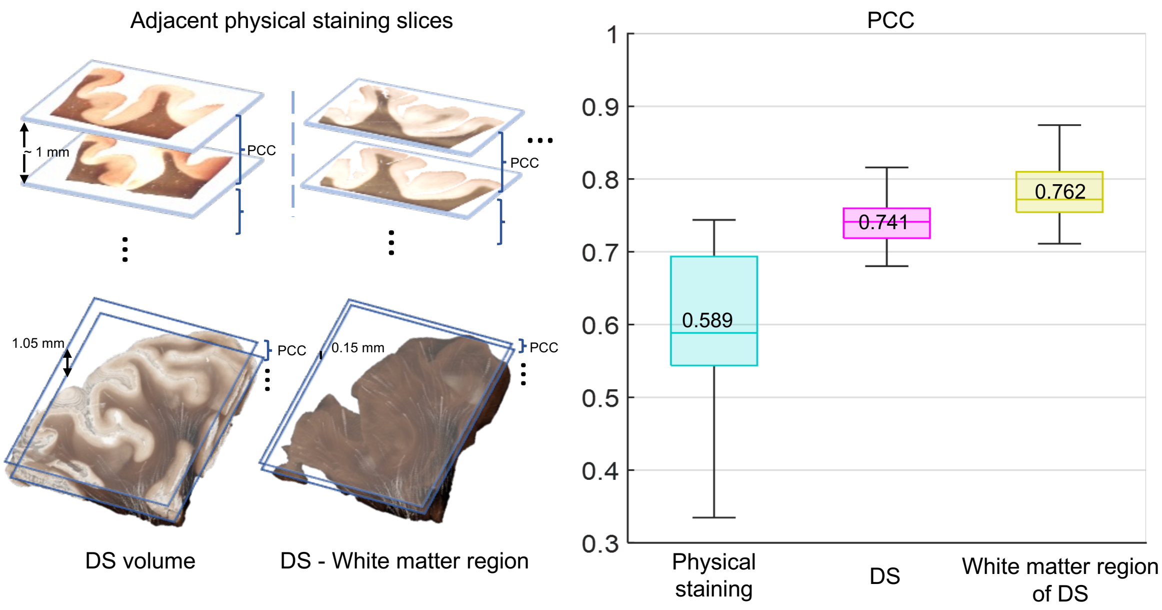

Figure S17. Quantification of volumetric geometry distortion on physical staining and our DS-OCT technique. On the left, Pearson correlation coefficients (PCC) are calculated on (1) each pair of adjacent physical staining slices of ~1 mm apart across the whole staining data set, (2) each pair of adjacent DS slices on the large unseen brain volume of 1.05 mm (7 slices) apart for fair comparison, (3) each pair of adjacent DS slices on the same volume of 0.15 mm (1 slice) apart within the segmented white matter region. PCC scores are aggregated and shown on the right.

16. Quantification of staining variability

A major advantage of DS compared PS is that DS significantly reduce staining variability. To support this argument, we quantify the staining variability on physical staining and our DS-OCT technique. Two perceptually relevant image metrics are shown across the whole testing dataset: average color saturation and intensity contrast. As annotated, staining slices (1), (2) and (3) are graded by our pathologist as over-stained, while (4), (5) and (6) are graded as under-stained. Those annotations align well with the outliers of the two quantitative metrics. The results are shown in Figure S18.

Here, average color saturation is defined by

$$\text{Saturation} = \frac{1}{N}\sum_{i=1}^{N}\frac{\sqrt{a_i^2 + b_i^2}}{L_i} \qquad (21)$$

where (L, a, b) is the CIELAB color space representation of the PS or DS image.

Intensity contrast is defined by the standard deviation of pixel intensities, given that $\boldsymbol{I}$ represents the normalized pixel intensity of the PS or DS image:

$$\text{Contrast} = \sqrt{\frac{1}{N}\sum_{i=1}^{N}(I_i - \bar{I})^2} \qquad (22)$$

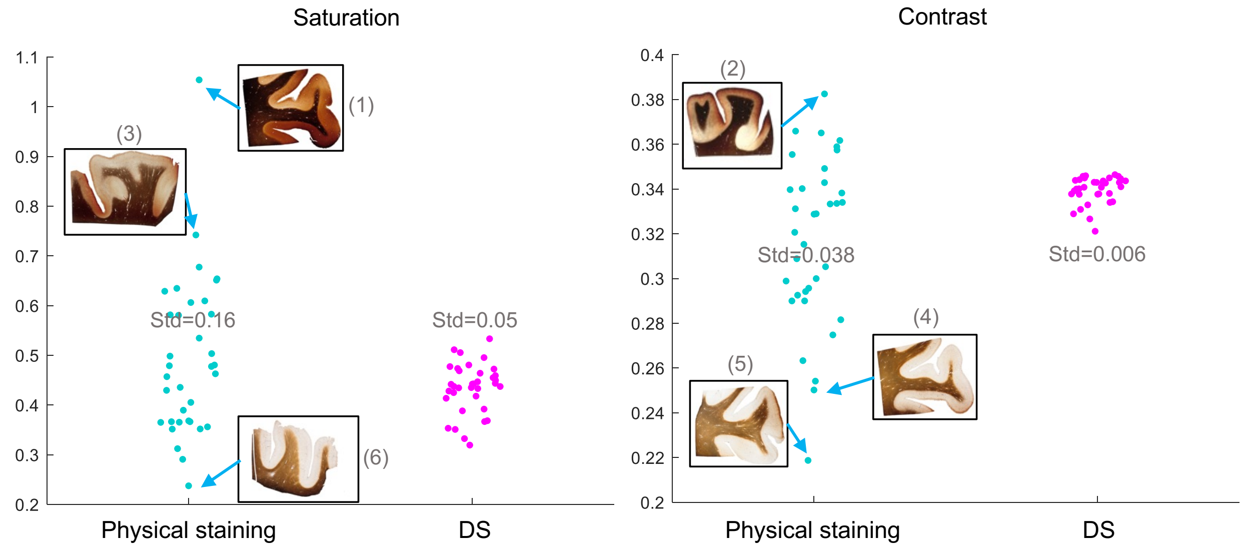

Figure S18. Quantification of staining variability on physical staining and our DS-OCT technique. Two perceptually relevant image metrics are shown across the whole testing dataset: average color saturation and intensity contrast. As annotated, staining slices (1), (2) and (3) are graded by our pathologist as over-stained, while (4), (5) and (6) are graded as under-stained. Those annotations align well with the outliers of the two quantitative metrics.

17. Independence of training and testing dataset

We provide an intuitive visualization of the data independence across our training and testing sets. The total 35 WSIs from 15 samples are divided into two groups: training groups (9 WSIs) and testing groups (26 WSIs). Each WSI is further cropped to non-overlapping 512 × 512 patches as input to the DS network G. We calculated the PCC score of each arbitrary pairs of WSIs and image patches across the whole training and testing dataset. As shown in Figure S19, the green, magenta and yellow boxes highlighted the correlation scores between any two images of the three cases, respectively: (1) both within the training group, (2) one from the testing while the other from the training group, (3) both within the testing group. The correlation matrix is shown on the left. On the right is the score distribution where each data point indicates the average correlation with respect to one image.

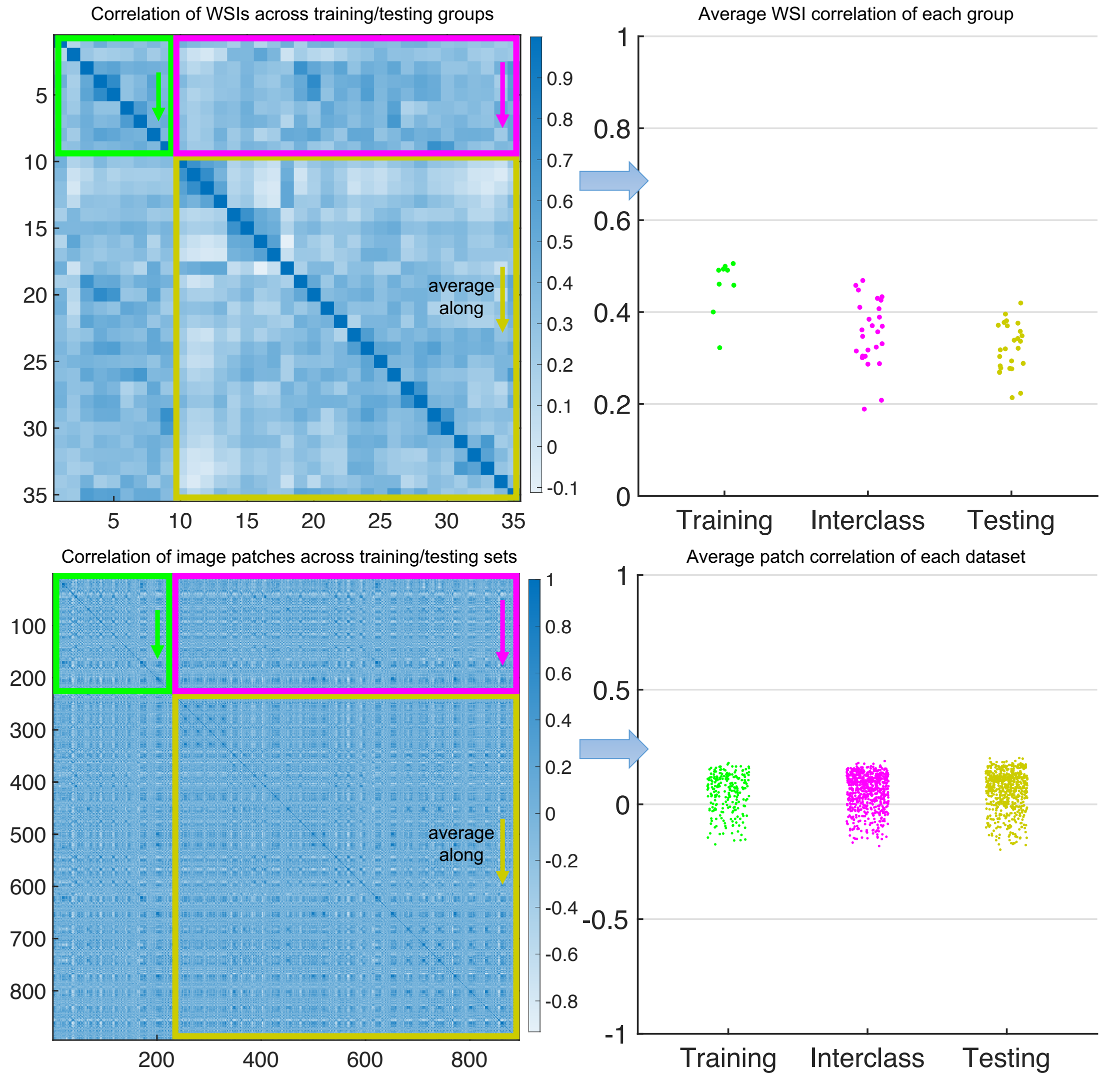

Figure S19. Data independence of training and testing datasets. PCC scores are calculated on arbitrary pairs of data across the whole dataset. WSI-scale correlation is shown at the top, and patch-scale correlation is shown at the bottom. Green, magenta and yellow colors indicate correlation scores of (1) intra-training-set, (2) interclass correlation between training and testing sets, (3) intra-testing-set. Correlation matrices and distribution are shown from left to right.

The distribution of correlation scores shows no visible difference across three cases. This indicates our data are evenly distributed across the training and testing datasets. We observed ~0.3 average WSI correlation in arbitrary pairs of the data either inter class or intra class, which has an offset above 0 due to global geometric similarity and the large white background common in the samples. Here, the geometric similarity is irrelevant to the independence of the data. As shown at the bottom of Figure S19, this offset is trivially near zero when the WSI images are cropped to image patches with no global similarity. Indeed, these image patches are the actual input data seen by our DS network *G* during training and testing. In either stage, the generator *G* is agnostic of the global geometry of the sample WSI and the subject where the sample comes from.

Reference:

1 Isola P, Zhu J-Y, Zhou T, Efros AA. Image-to-Image Translation with Conditional Adversarial Networks. In: *2017 IEEE Conference on Computer Vision and Pattern Recognition (CVPR)*. IEEE: Honolulu, HI, 2017, pp 5967–5976.

2 Zhu J-Y, Park T, Isola P, Efros AA. Unpaired Image-To-Image Translation Using Cycle-Consistent Adversarial Networks. In: *Proceedings of the IEEE International Conference on Computer Vision*. 2017, pp 2223–2232.

3 Park T, Efros AA, Zhang R, Zhu J-Y. Contrastive Learning for Unpaired Image-to-Image Translation. In: Vedaldi A, Bischof H, Brox T, Frahm J-M (eds). *Computer Vision – ECCV 2020*. Springer International Publishing: Cham, 2020, pp 319–345.

4 Chang S, Varadarajan D, Yang J, Chen IA, Kura S, Magnain C *et al.* Scalable mapping of myelin and neuron density in the human brain with micrometer resolution. *Sci Rep* 2022; **12**: 363.

5 Reinhard E, Adhikhmin M, Gooch B, Shirley P. Color transfer between images. *IEEE Computer Graphics and Applications* 2001; **21**: 34–41.

6 Schneider CA, Rasband WS, Eliceiri KW. NIH Image to ImageJ: 25 years of image analysis. *Nat Methods* 2012; **9**: 671–675.

7 Walt S van der, Schönberger JL, Nunez-Iglesias J, Boulogne F, Warner JD, Yager N *et al.* scikit-image: image processing in Python. *PeerJ* 2014; **2**: e453.
